# Analysis of Soluble Molecular Fibronectin-Fibrin Complexes and EDA-Fibronectin Concentration in Plasma of Patients with Atherosclerosis

**DOI:** 10.1007/s10753-016-0336-0

**Published:** 2016-03-29

**Authors:** Anna Lemańska-Perek, Dorota Krzyżanowska-Gołąb, Małgorzata Pupek, Piotr Klimeczek, Wojciech Witkiewicz, Iwona Kątnik-Prastowska

**Affiliations:** Department of Chemistry and Immunochemistry, Medical University of Wrocław, Bujwida 44a, 50-345 Wrocław, Poland; Provincial Specialist Hospital in Wrocław, Research and Development Center in Wrocław, Wrocław, Poland

**Keywords:** fibronectin, fibronectin bearing EDA segment, FN-fibrin complexes, atherosclerosis

## Abstract

Atherosclerosis, a chronic vascular disease, leads to molecular events bound with interplaying processes of inflammation and coagulation. In the present study, fibronectin (FN), FN containing extra domain A (EDA-FN), frequency of occurrence, and relative amounts of soluble plasma FN-fibrin complexes were analyzed in 80 plasma samples of patients suspected of coronary artery disease based on clinical evaluation and changes in arteries found by computed tomographic coronary angiography. The study showed that in the plasma of the patients’ group with high risk of coronary artery disease EDA-FN concentration was significantly higher (3.5 ± 2.5 mg/L; *P* < 0.025) and the molecular FN-fibrin complexes of 1000 kDa and higher occurred more often than in the groups of patients with mild risk of coronary artery disease and the normal age-matched. The increased level of EDA-FN and occurrence of FN-fibrin complexes could have a potential diagnostic value in the diagnosis and management of patients with coronary artery disease.

## INTRODUCTION

Development of atherosclerotic lesions, which increasingly affect the human population, causing high morbidity and mortality, are known to be associated with chronic inflammation and can lead to thrombosis and diverse cardiovascular diseases [1]. At the cellular and molecular levels, atherosclerosis is known to activate immune-inflammatory pathways, encompassing multiple complex interdependent interactions among inflammatory cells, vascular elements, extracellular matrix molecules, and plasma proteins through expression of cytokines, and several adhesion molecules and their receptors [2–4]. The atheroprogression is manifested by the development of atheromatous plaque in the affected vessel wall, which leads to diverse vascular lesions and disturbs molecular vessel functions, vascular permeability, and flow of blood around the body [2, 3, 5].

A key event associated with plaque formation is expansion and remodeling of extracellular matrix components (ECM) in susceptible arterial areas [6].

During atherosclerotic lesions under disease-dependent conditions, the normal complex organization of ECM undergoes harmful structural and functional modifications which can lead to the occurrence of numerous diseases [6–8]. Among the major proteins of ECM responsible for the organization and regulation of ECM-dependent molecular functions, a multifunctional and multidomain glycoprotein, fibronectin (FN), plays a crucial role [8–10]. FN, besides being an insoluble component of ECM and tissues (cFN), is also an abundant protein of plasma and other physiological fluids. In plasma and tissues, FN can exist in diverse isoforms arising from posttranslational modifications (N- and O-glycosylation, phosphorylation), alternative splicing of FN pre-messenger RNA (i.e., inclusion of EDA, EDB, and IIICS segments), and moreover from variable conformations depending on environmental conditions (globular and fibrillar structures) [8, 9, 11].

FN is a large dimeric glycoprotein (450–500 kDa); each monomer of which consists of types I, II, and III of repeating amino acid units. The multiple copies of repeats are arranged into several domains able to bind fibrin, collagen, glycosaminoglycans, and cellular receptors [9, 10]. Two identical or nearly identical (depending on the included or excluded spliced extra domains) FN polypeptides are linked together by two disulfide bonds near their carboxyl termini. In contrast, their N-termini ends are unlinked, facilitating formation of many diverse conformational structures able to react with FN ligands [9, 11]. Its hepatic origin, plasma form of FN (pFN) lacking extra EDA and EDB segments, has a looped compact conformation which can be stretched to an unfolded conformation when FN is caught by cellular integrin receptors [9, 11]. The tissue cellular FN (cFN) is synthesized by many cell types (e.g., fibroblasts, endothelial cells, platelets, and monocytes) and bears variable proportions of EDA and EDB segments (EDA-FN and EDB-FN, respectively) [9, 12].

Both forms, pFN and cFN, are reported to be incorporated into the fibrillar network of ECM [9, 10, 13], where they play structural and functional roles regulating some cellular activities [9, 14]. The plasma-derived FN is reported to support hemostasis, regulate thrombosis [15, 16], and significantly accelerate healing, reducing the area of inflammation [17, 18]. Plasma FN is also a major component of the blood clot. With fibrin and its degraded forms, it readily forms a macromolecular complex which can be cross-linked covalently in the reaction catalyzed by transglutaminase XIIIa [16, 19]. Through binding with fibrin, pFN influences the rate of formation, stability, as well as the structure of the fibrin matrix [15, 20].

In spite of the fact that both EDA- and EDB-FN isoforms take part in vasculogenesis in embryos and angiogenesis in cancer and non-tumoral conditions, the specific integrin receptors have been identified exclusively for EDA-FN [12, 21, 22]. FN carrying the EDA segment recognized by integrin receptors is reported to be implicated in efficient adhesion, activation, and aggregation of platelets, promoting in that way inflammation and coagulation processes [23–25]. The initial step of platelet adhesion is immobilization of dimeric EDA-FN on platelets by integrins α5β1 and αIIbβ3 and stretching of a dimeric cFN to its fibrillar form. Maurer *et al.* [2015] provided experimental evidence that a fibrillar form of EDA-FN is a potent thrombogenic component of the subendothelium. The interaction of immobilized fibrillary EDA-FN to platelet integrins together with glycoprotein Ib-V-IX complex, a major signaling receptor for collagen, and Toll-like receptor 4 initiates molecular reactions which lead to activation of the coagulation cascade and promotion of thrombus formation [23]. These events bring a high risk of atherosclerosis and thrombosis development [16, 26–28].

On the other hand, EDA-FN located on the top of a plaque is known to form a protective fibrous cap, which prevents plaque rupture and vascular occlusion [26, 29–31].

In the present study, frequency of occurrence and relative amounts of soluble plasma FN-fibrin complexes were determined in plasma of patients suspected of coronary artery disease based on significant changes observed in the patients’ artery. The relative amounts of soluble plasma FN-fibrin complexes were analyzed with respect to FN and EDA-FN concentrations, occurrence of FN monomer, and eventual presence of FN fragments. The FN and EDA-FN concentrations were estimated by ELISA with two specific monoclonal antibodies, and the occurrence and relative amounts of FN-fibrin complexes and FN monomer and possible presence of FN degradation products were analyzed by SDS-agarose immunoblotting [32] and Western blotting [33], respectively. The study indicates that the analysis of FN-fibrin complexes and the EDA-FN concentration can help to better understand the mechanisms underlying thrombosis and changes associated with endothelial dysfunction and vascular diseases.

## MATERIALS AND METHODS

### Patients

Patients were included in the study after their clinical evaluation was performed and informed consent had been given. The study was approved by the Bioethical Committee at the Regional Specialist Hospital in Wroclaw and complies with the 1975 Declaration of Helsinki.

Participants (*n* = 80, 39 women and 41 men, aged 63.8 ± 9 years) were recruited to the study from persons attending the Emergency Department of the Regional Specialized Hospital in Wroclaw because of chest pain and inconclusive unstable angina pectoris detection by classical tests such as high-sensitive troponin I (hsTnI) and electrocardiography (ECG).

After hospitalization, the patients suspected of coronary artery disease (CAD), but with myocardial infarction excluded, were included in the study based on standard computed tomographic coronary angiography (CTCA) performed as describe previously by Miszalski-Jamka *et al.* [34]. CTCA was performed using a 2×32-slice Dual Source Computed Tomography Scanner (Somatom Definition, Siemens, Erlangen, Germany). Images were reconstructed using a B26f kernel with an image matrix of 512 × 512 pixels. A multiphase reconstruction (from 0 to 90 %) was performed and the best quality image reconstructions were assessed. The post processing and study evaluation were performed using a dedicated workstation (3D Leonardo, Syngo Via Siemens Medical Solution, Erlangen, Germany).

### Sampling

Blood samples (4.5 ml), anti-coagulated with 3.2 % sodium citrate (0.5 ml), were collected from 80 patients suspected of CAD and plasma was immediately separated from the blood cells by centrifugation at 2000×*g* for 10 min. The samples were aliquoted and stored at −76 °C until analysis.

Eighty patients’ samples were divided into groups 1 and 2 according to the degree of vessel lumen narrowing caused by arteriosclerotic plaque distinguished based on the data of computed tomographic coronary angiography [34]. Group 1 with significant coronary artery changes comprised 66 plasma samples of patients (30 females and 36 males; mean age 64.4 ± 9 years) with presence of atherosclerotic plaques in the coronary arteries and with one or more visible stenosis (>50 % vessel lumen narrowing). Group 2 with mild coronary artery changes comprised 14 blood plasma samples of patients (9 females and 5 males; mean age 61.5 ± 9 years) with (<50 % vessel lumen narrowing) or without visible vessel lumen narrowing. The characteristics of the blood parameters of patients are summarized in Table 1.

**Table 1:** Biochemical Characteristics of the Groups of Patients with Atherosclerosis

Parameter	Groups			
Number of samples female/male age, years	1. Significant coronary artery changes *n* = 66 30/36 64.4 ± 9		2. Mild coronary artery changes *n* = 14 9/5 61.5 ± 9	
Total cholesterol mg/dL	200.5 ± 50.7 195 (162–237) 30↑		198.2 ± 39.2 204 (167–212) 7↑	
HDL mg/dL	Female 56.8 ± 26.7 52.5 (46–64) 7↓	Male 59.4 ± 49.1 45 (40–53)	Female 55.3 ± 13.4 51.5 (46.5–62.5) 2↓	Male 52.6 ± 31.3 41 (36–45) 2↓
LDL mg/dL	122.1 ± 45 118 (82–153) 40↑		115.9 ± 22.2 107 (105–119) 10↑	
BMI kg/m^2^	26.6 ± 5.7 27.7 (24.1–29.85)		26.6 ± 8.1 26 (25.4–32)	
Glucose mg/dL	108.6 ± 34.3 104 (94–115) 36↑		130.5 ± 50.4 109 (97–175) 10↑	
CRP mg/L	3.6 ± 3.9 2 (0.95–5.7) 16↑		4.6 ± 7.4 2.15 (0.97–4.55) 3↑	
hsTnI ng/ml	<0.04 (0.09 ± 0.06) 11↑		<0.04	

Values of blood parameters are given as mean values ± SD, median, and (25th and 75th) quartiles. ↑↓ the number of samples with low (↓) or high (↑) value. All parameters were determined using routine laboratory techniques. All biochemical parameters were determined with commercial kits, and CRP levels were measured with a coagulometer

*HDL* high-density lipoprotein, *LDL* low-density lipoprotein, *BMI* body mass index, *CRP* C-reactive protein, *hsTnI* high-sensitive troponin I (TnI was measured twice, at the time of admission to an emergency department and 6 h after)

The control group comprised samples taken from healthy non-smoking volunteers (13 females and 8 males; mean age 65.3 ± 12 years), members of the Wrocław Medical University staff, and 8 people who visited the emergency department because they felt a pain in the chest, but based on the results of CTCA and normal values of routine laboratory blood parameters were included in the control group.

### FN Concentration

Plasma FN (pFN, test 1) and EDA-FN (test 2) concentrations were determined by two independent ELISAs using a well-defined domain-specific monoclonal antibody directed to a centrally located comprehensive cell-binding domain of FN (FN30-8; M010 TaKaRa Shuzo Co. Ltd., Shiga, Japan) [32] or to an extra domain EDA of FN (DH1, EMD Millipore, Merck KGaA Darmstadt, Germany). The monoclonal antibodies anti-FN (test 1) and anti-EDA-FN (test 2), diluted 1:10 000 and 1:3000, respectively, in TBS, were used as a coating agent in the wells of a microtiter plate (Nalge Nunc International, Naperville, IL, USA) to bind FN and EDA-FN in the samples. The amount of FN bound by the monoclonal antibody was quantified by rabbit anti-FN polyclonal antibodies (Sigma Chemical Co, St Louis, MO, USA, diluted 1:5000 (test 1), or 1:2500 (test 2) in TBS containing 0.1 % Tween-20) and peroxidase-conjugated goat anti-rabbit immunoglobulins (Sigma Chemical Co, St Louis, MO, USA), diluted 1:20,000 (test 1), or 1:3000 (test 2) as the secondary antibodies. The amount of FN was assayed by a colorimetric reaction using o-phenylenediamine dihydrochloride/H_2_O_2_ as the enzyme substrate and measured in a Stat Fax 2100 Microplate Reader (Awareness Technology Inc, Palm City, FL, USA) at 450 nm with 630 nm as a reference filter. The samples were analyzed in two different sample dilutions, each in duplicate. The pFN and EDA-FN concentrations are given in milligram per liter and presented as the mean ± standard deviation (SD).

To determine non-specific binding, two controls were included in the tests: minus primary antibody and minus secondary antibody.

A human plasma FN preparation (Sigma, St. Louis, MO, USA, from 2.5 to 50 ng/well) was used as a standard for FN-ELISA determination (test 1), and acellular fibronectin from human foreskin fibroblasts (Sigma, St. Louis, MO, USA, from 3.125 to 50 ng/well) for EDA-FN-ELISA (test 2).

### Western Immunoblotting

Plasma samples containing 300 ng of FN were subjected to SDS-(10 %) polyacrylamide gel electrophoresis under reducing conditions as described earlier [35].

### SDS-Agarose FN Immunoblotting

Plasma FN-fibrin complexes were revealed by SDS-agarose immunoblotting as described previously [32].

### Statistics

Data are presented as means ± standard deviations (SD). Comparisons between groups were performed by means of the Kruskal–Wallis and *post hoc* tests. *P* values less than 0.05 were regarded as significant.

Receiver operating characteristic (ROC) analysis was performed with R for Windows (the R Foundation for Statistical Computing, Vienna, Austria).

To estimate the usefulness of the FN-fibrin complexes and EDA-FN for differential diagnostics of the state of patients with significant coronary artery changes compared to mild coronary artery changes, statistic cluster analysis was used (Statistica version 12 PL, StatSoft Inc., Tulsa, OK, USA). The results are presented as a dendrogram, starting with all the subjects (patients and controls) in one cluster, finally leading to separate clusters as leaves of a binary tree.

## RESULTS

### Plasma FN Concentration

**Fig. 1:**
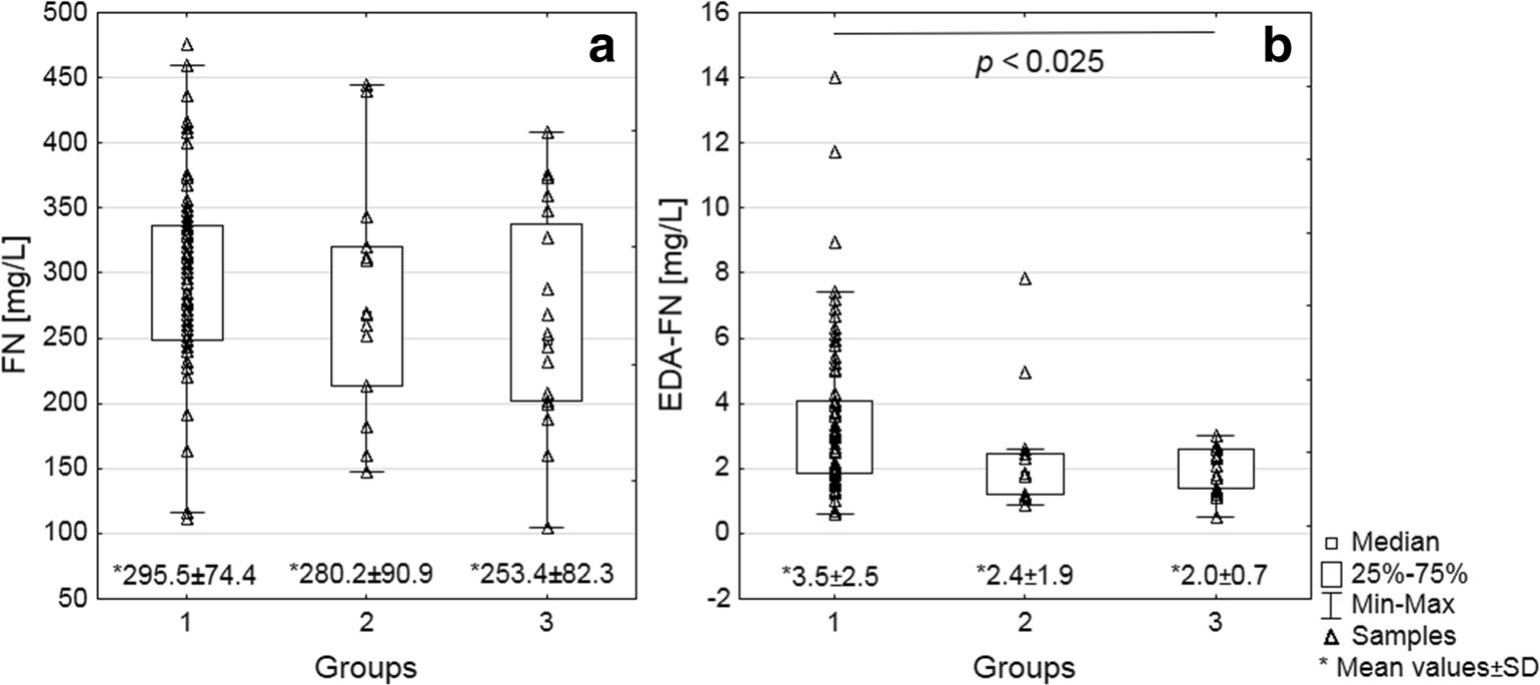
Distribution of FN (**a**) and EDA-FN (**b**) concentrations in plasma of patients with atherosclerosis. pFN (**a**) and EDA-FN (**b**) concentrations were determined by ELISA [35], using two monoclonal antibodies domain-specific directed to cell-binding domain of FN (FN30-8; M010 TaKaRa Shuzo Co. Ltd., Shiga, Japan) and EDA segment of FN (EMD Millipore, Merck KGaA Darmstadt, Germany). For details see “Materials and Methods.” Data are given as mean values ± SD, median, and (25th and 75th) quartiles. Significantly different from the age-matched normal group calculated by the Kruskal–Wallis and post hoc tests.

The mean values of pFN concentration (Fig. 1a) were similar in patient group 1 with significant coronary artery changes (292.53 ± 74.4 mg/L) and group 2 with mild coronary artery changes (280.21 ± 90.9 mg/L), but slightly higher than in the control group (253.38 ± 82.3 mg/L).

### EDA-FN Concentration

Compared with the control group (2.0 ± 0.7 mg/L), the mean EDA-FN concentration (Fig. 1b) was significantly higher in group 1 (3.5 ± 2.5 mg/L; *P* < 0.025) and at a similar level in group 2 (2.4 ± 1.9 mg/L).

The ROC curve analysis of EDA-FN concentration (Fig. 3a) showed a sensitivity of 53 % and specificity of 91 % (area under the curve [AUC] 0.67).

### FN Fragments

The electrophoresis of plasma samples in 10 % polyacrylamide gel under reducing conditions and subsequent immunoblotting with anti-FN monoclonal antibody did not show the presence of FN fragments.

### Occurrence of FN-Fibrin Complexes

**Fig. 2:**
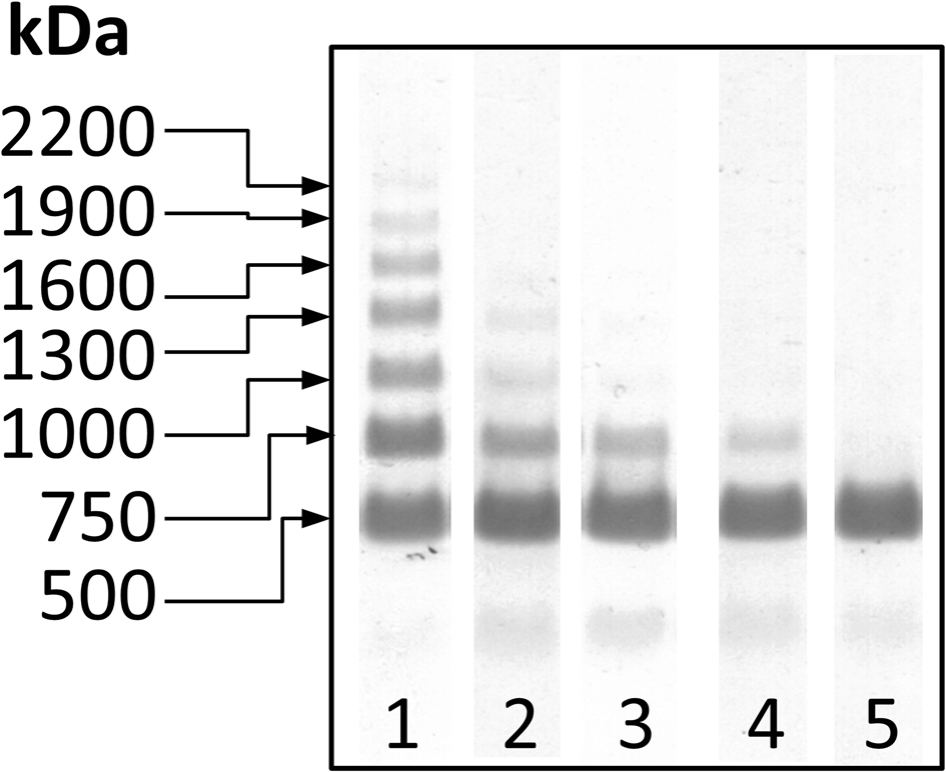
Representative immunopatterns of FN-fibrin complexes in plasma samples of patients with atherosclerosis and age-matched normal group. The 101 blood plasma samples of atherosclerotic patients and age-matched normal individuals were subjected to SDS-agarose immunoblotting under non-reducing conditions [32]. For details see “Materials and Methods.” Plasma samples: *lanes 1*–*2*, significant coronary artery changes; *lane 3*, mild coronary artery changes; *lanes 4*–*5*, normal age-matched plasma. The molecular masses of the 750 to 2200 kDa plasma FN-fibrin complexes and 500 kDa FN dimer are shown by *arrows* on the left.

SDS-agarose immunoblotting revealed the presence, beside bands corresponding to FN dimer (500 kDa) and FN monomer (∼250 kDa), of FN-fibrin bands with decreasing electrophoretic mobilities (Table 2, Fig. 2) and increasing molecular masses of 750, 1000, 1300, 1600, 1900, and 2200 kDa. The FN-fibrin bands were numbered as FN-fibrin complexes I–VI, respectively.

**Table 2:** Frequency of Occurrence and Relative Amount of Fn-Fibrin Complexes in Plasma of Patients with Atherosclerosis

Plasma FN forms	*M*_m_ (kDa)	Frequency of occurrence and relative amount of FN forms in plasma groups 1–3 Mean value of relative amount ± SD		
		1. Significant coronary artery changes *n* = 66	2. Mild coronary artery changes *n* = 14	3. Age-matched normal group *n* = 21
FN monomer ± degradations fragments	∼250	0.94 (62) 6.24 ± 3.4	0.92 (14) 6.0 ± 3.2	0.85 (18) 4.95 ± 4.5
FN dimer	∼500	1 (66) 74.66 ± 17.7	1 (14) 86.33 ± 8.5	1 (21) 88.13 ± 7.5
FN-fibrin complexes	I ∼750	0.83 (55) 13.36 ± 10.2 *P* < 0.048	0.64 (9) 7.25 ± 6.7	0.76 (16) 6.88 ± 4.6
	II ∼1000	0.39 (26) 2.95 ± 4.6 *P* < 0.019	0.07 (1) 0.19 ± 0.7 ^a^2.7	Not detected
	III ∼1300	0.28 (19) 1.62 ± 3.3	Not Detected	Not Detected
	IV ∼1600	0.2 (13) 0.77 ± 2.1	Not detected	Not detected
	V ∼1900	0.05 (3) 0.21 ± 1.0	Not detected	Not detected
	VI ∼2200	0.03 (2) 0.08 ± 0.6	Not detected	Not detected

Plasma FN forms were revealed by SDS-agarose immunoblotting (see Fig. 2). Frequency of occurrence is the ratio of the number of samples containing the FN form to the total number of samples. In parentheses are given the number of samples which revealed the respective FN band. The relative amount of the FN band is the percentage of the total number of pixels found in the electrophoresis path and is expressed as mean value ± SD. Data are presented as mean values ± SD. Significantly different from the age-matched normal group calculated by the Kruskal–Wallis and post hoc tests

^a^The relative amount for the sample from group 2 with mild coronary artery changes (∼1000 kDa)

The plasmas of group 1 (significant changes in the coronary artery) contained the set of FN-fibrin complexes I–VI whose frequency of occurrence (from 0.83 for complex I to 0.03 for complex VI) and their relative amounts (from 28.3 % for complex I to 4.4 % for complex VI) decreased with the increased molecular mass of the FN-fibrin complexes. In contrast, the plasma of group 2 (mild coronary artery changes) and age-matched controls, beside the FN-fibrin complex I occurring in about 70 % of samples, contained none of the detectable FN-fibrin complexes II–VI, excluding one sample of group 2, which revealed the presence of complex II. Moreover, the mean relative amount of FN-fibrin complex I (13.36 ± 10.2 %) was higher in group 1 than in group 2 (7.25 ± 6.7 %) and the control group (6.88 ± 4.6 %).

**Fig. 3:**
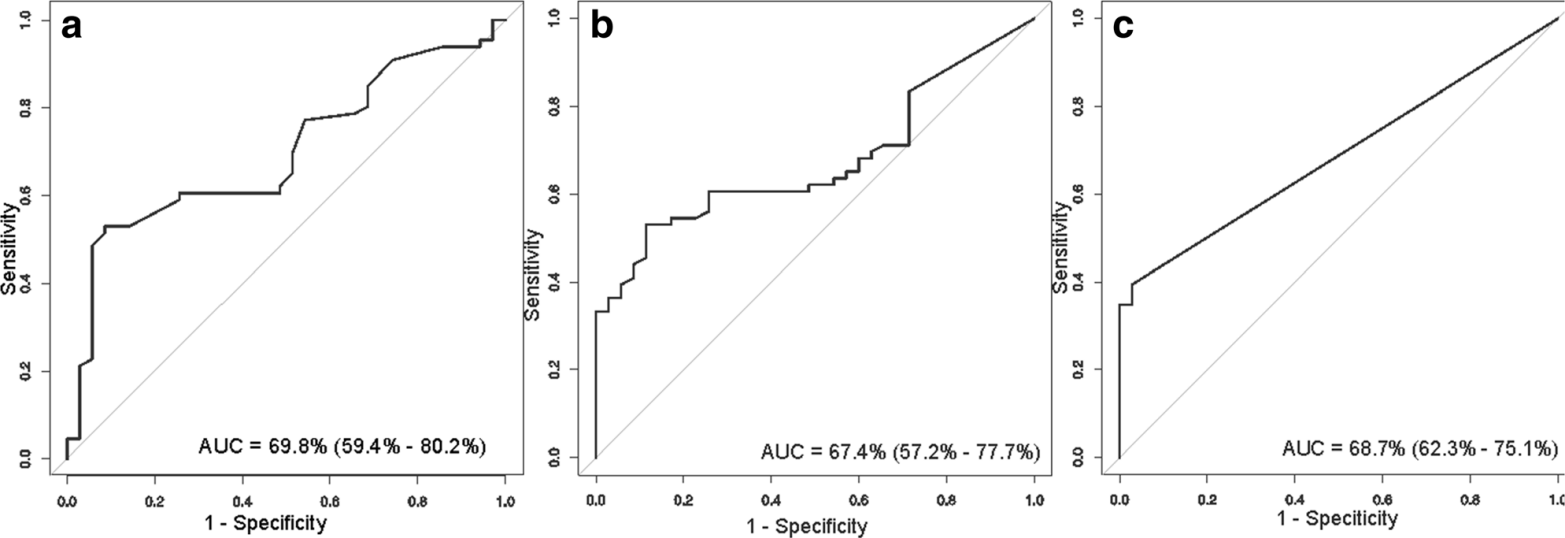
ROC curves for values of EDA-FN concentration (**a**) and FN-fibrin complexes I (**b**) and II (**c**) relative amounts in plasma of patients with atherosclerosis. Data are given as AUC with 95 % confidence interval.

ROC curve analysis of the relative amounts of FN-fibrin complexes (∼750 and ∼1000 kDa) identified parameters with a sensitivity of 53 % and specificity of 88 % (AUC 0.67) for complex I (Fig. 3b) and a sensitivity of 39 % and specificity of 97 % (AUC 0.68) for complex II (Fig. 3c).

**Fig. 4:**
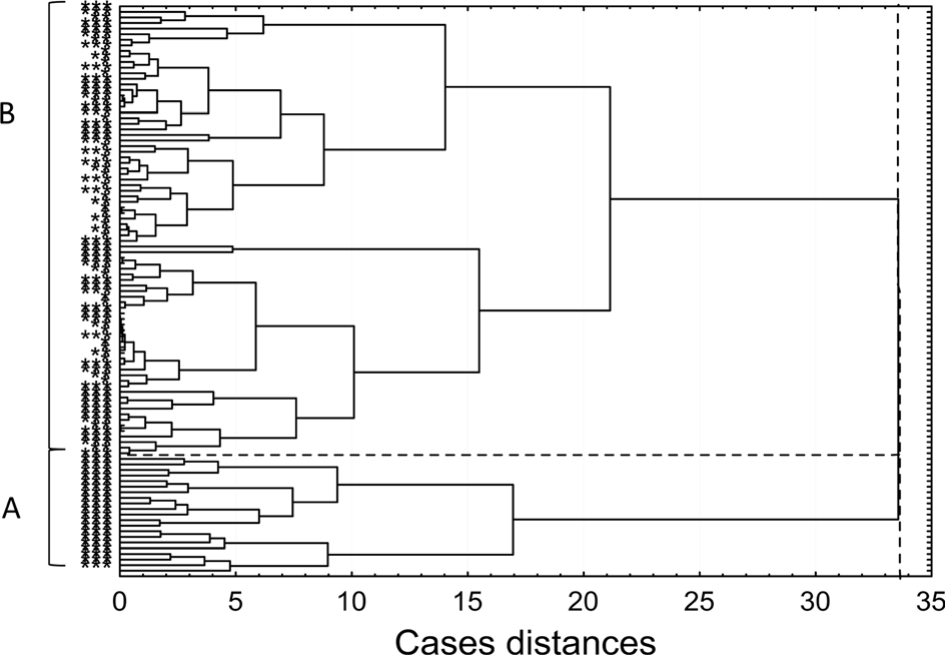
Dendrogram of cluster analysis based on the data of FN-fibrin complexes and EDA-FN levels of human blood plasma samples of atherosclerotic patients and age-matched normal individuals. *Triple asterisks indicate* significant coronary artery changes, *double asterisks* mild coronary artery changes, and *asterisk* normal age-matched plasma. *Cluster A* and cut-off lane governing patients with significant coronary artery changes from the rest of samples with significant or mild coronary artery changes and controls (*cluster B*).

Cluster analysis (Fig. 4) based on the relative amounts of FN-fibrin complexes and EDA-FN concentration data of groups 1, 2, and the age-matched group allowed the cluster A samples (*n* = 21, 32 %) from group 1 to be separated from the rest of the samples (cluster B).

The analysis for atherosclerosis risk factors (total cholesterol, LDL, HDL, BMI, CRP, glucose level, sex, and age) showed that the samples from cluster A have slightly higher levels of HDL and LDL (62.2 ± 50.4 and 130.6 ± 45.9, respectively) compared to the rest of the patients from the group of significant coronary artery changes (56.1 ± 33.8 and 117.8 ± 44.4, respectively). Moreover, the patients in this subgroup were significantly younger (mean age 60.4 ± 8 years, *P* < 0.022) than the rest of patients in this group (mean age 66 ± 9 years).

## DISCUSSION

The study showed that the presence of soluble FN-fibrin complexes and high concentration of the FN isoform bearing an EDA segment (EDA-FN) in patients’ plasma were associated with significant changes in the coronary artery, and these subjects were suspected of high risk of coronary artery disease (CAD) more often than in those with mild or no changes in blood vessels.

The high EDA-FN concentration (Fig. 2b) in the plasma samples of patients with high risk of CAD compared with its low level in the plasma of controls as well as patients with low risk of CAD (Fig. 2b) has also been observed by others in ischemic stroke [36], diabetes [37], atherosclerosis [30], and cancer [38]. EDA-FN occurrence in plasma at a high concentration is believed to be associated with inflammation and its release from tissues to the circulation during arterial wall lesions in thrombosis [39], plaque formation, development and progression of atherosclerosis, and arterial aging [24, 26–28]. However, the high EDA level in plasma can also reflect other pathophysiological processes connected with reconstruction of ECM [37] which can mask the changes due to atherosclerosis [30]. On the other hand, pFN (lacking an EDA segment) level in the plasma samples of patients with high and low risk of CAD did not differ from that in the normal group (Fig. 1a), and its diagnostic usefulness has been reported to be controversial, being probably due to different exclusion criteria for patients and differential timing of FN measurements in relationships to disease [16].

The present work also revealed the presence of FN-fibrin complexes of 750, 1000, 1300, and 1600 kDa in some plasma samples of patients with a high risk of CAD (Table 2, Fig. 2), which were absent in the plasma of age-matched patients and those with a low risk of CAD, though with the exception of the presence of the complex with the smallest molecular mass of 750 kDa. The presence of the set of the FN-fibrin complexes has been previously reported by our group for plasma of patients with some inflammatory diseases [32, 40]. In contrast, none of them were found in plasma of young and middle aged healthy individuals, but the 750-kDa complex occurred progressively with aging of individuals [32, 33, 41].

**Fig. 5:**
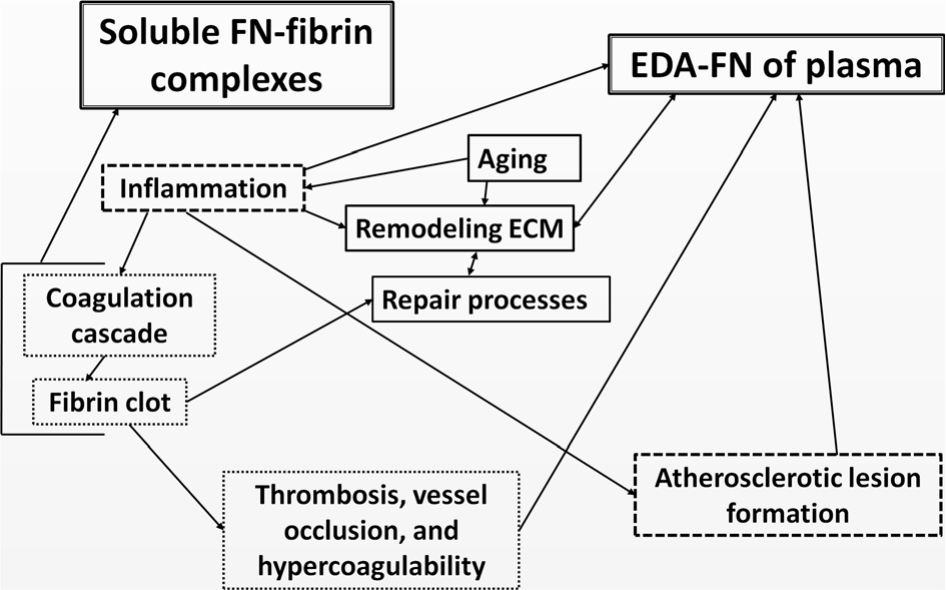
The role of EDA-FN and FN-fibrin complexes in atherosclerosis. The released inflammatory agents during a disease are known to initiate the reactions of the coagulation cascade and formation of FN-fibrin complexes, which can be easily detected by agarose immunoblotting as a ladder of bands showing molecular masses from 750 to 2200 kDa (Fig. 2). The FN-fibrin complexes occurring in plasma may lead to hypercoagulability, thrombosis, and vessel occlusion [15, 42]. Moreover, the harmful inflammatory agents and age-related processes occurring in arteries provoke ECM remodeling [43], which results from release of ECM components including cellular FN bearing an EDA segment (Fig. 1b). EDA is known to play a significant role in the repair process and in atherosclerosis [9, 26–28].

The occurrence of FN-fibrin complexes in plasma of patients seems to reflect molecular events connected with interplaying processes of inflammation, immunity, and coagulation (Fig. 5). When the coagulation process is activated by inflammatory agent/s, the plasma fibrinogen is converted to fibrin, which in contrast to untouched fibrinogen is able to form a complex with pFN. The fibrils of FN-fibrin are rapidly deposited in the injured vessel wall as the first factor in the wave of hemostasis and clot formation. The cascade of reactions of the coagulation system is under control and the thrombus is dissolved by the fibrinolytic system. In consequence, the lesion in the blood vessel is repaired by the wound healing process [9]. However, any imbalance/dysregulation between procoagulant/antifibrinolytic and anticoagulant factors may lead to hypercoagulation, destruction of vessels, thrombosis, and atherosclerosis (Fig. 3). Such a situation may happen in disease-dependent conditions and with aging [44].

The FN-fibrin complexes are reported to play a different biological role than unbound pFN [15]. The pFN-fibrin complex supports platelet aggregation and thrombosis and has prothrombotic activities [15, 42]. Moreover, Wang *et al.* (2014) reported that pFN through interactions with fibrin is able to switch from supporting hemostasis to inhibiting thrombosis/vessel occlusion, depending on the gradient of fibrin, and indicate that fibronectin is a self-limiting regulator in thrombosis [15].

Interestingly, the relative amounts of the FN-fibrin complexes did not show any correlation with the plasma EDA-FN concentration. The high concentration of EDA-FN probably reflects progression of the atherogenic lesion, whereas the presence of FN-fibrin complexes in high amounts contributes to the hypercoagulation state (Fig. 5), although to a variable degree. The profile of FN-fibrin macro-complexes composed of the ladder of bands with molecular masses higher than 1000 kDa, not observed in the normal plasma or in the samples of patients with mild coronary artery changes, but evidently revealed in the plasma of patients with significant coronary artery changes (Table 2), might be related to increased coagulation. The patients whose plasma revealed such a pattern of FN-fibrin complexes might be suspected of hypercoagulability and high risk of thrombosis, and they should be under special medical care. However, it could not be excluded that the presence of high-molecular FN-fibrin complexes might be bound additionally with severity of coexisting diseases. For example, we have observed the presence of the FN-fibrin complexes with molecular masses up to 1900 kDa in the plasma samples of patients with significant coronary artery changes and with acute chest pain, but without medical evidence of acute myocardial infarction. The cluster analysis based on the data of FN-fibrin complexes and EDA-FN levels (Fig. 4) allowed us to separate the subgroup of patients with significant coronary artery changes who should be under special medical care because of high risk of hypercoagulation and thrombosis.

To conclude, the profile analysis of FN-fibrin complexes and the determination of EDA-FN concentration, but not plasma FN in the plasma of patients, could be considered as a potential diagnostic biomarker helpful in diagnosis, management, and prophylaxis of patients with high risk of atherosclerosis and moreover may help to discriminate the group of patients with high risk of CAD from that with mild risk of CAD.
